# Multiplex Bead Array Assay for Detection of 25 Soluble Cytokines in Blister Fluid of Patients with Complex Regional Pain Syndrome Type 1

**DOI:** 10.1155/MI/2006/28398

**Published:** 2006-02-22

**Authors:** Claudia Heijmans-Antonissen, Feikje Wesseldijk, Renate JM Munnikes, Frank JPM Huygen, Patrick van der Meijden, Wim C. J. Hop, Herbert Hooijkaas, Freek J. Zijlstra

**Affiliations:** ^1^Department of Anesthesiology, Erasmus MC, P.O. Box 2040, 3000 CA Rotterdam, The Netherlands; ^2^Applied Cytometry Systems, Sheffield 8252JZ, Dinnington, UK; ^3^Department of Epidemiology & Biostatistics, Erasmus MC, P.O. Box 1738, 3000 DR Rotterdam, The Netherlands; ^4^Department of Immunology, Erasmus MC, P.O. Box 1738, 3000 DR Rotterdam, The Netherlands

## Abstract

Inflammatory processes are known to be involved at least in the early phase of complex regional pain syndrome type 1 (CRPS1). Blister fluid obtained from the involved extremities displayed increased amounts of proinflammatory cytokines IL-6 and TNFα compared with the noninvolved extremities. The aim of this paper is to investigate the involvement of mediators by measurement of several other cytokines using new detection techniques that enable multiple cytokine measurement in small samples. The use of a multiplex-25 bead array cytokine assay and Luminex technology enabled simultaneous measurement of representative (1) proinflammatory cytokines such as GM-CSF, IL-1β, IL-1RA, IL-6, IL-8, and TNF-α; (2) Th1/Th2 distinguishing cytokines IFN-γ, IL-2, IL-2R, IL-4, IL-5, and IL-10; (3) nonspecific acting cytokines IFN-α, IL-7, IL-12p40/p70, IL-13, IL-15, and IL-17; and (4) chemokines eotaxin, IP-10, MCP-1, MIP-1α, MIP-1β, MIG, and RANTES. Although minimal detection levels are significantly higher in the bead array system than those in common ELISA assays, in blister fluid, IL-1RA, IL-6, IL-8, TNF-α, IL-12p40/p70, MCP-1, and MIP-1β were detectable and increased in CRPS1 affected extremities. Levels of IL-6 and TNF-α simultaneously measured by ELISA (Sanquin Compact kit) and by multiplex-25 bead array assay (Biosource) were highly correlated (*r* = 0.85, *P* < .001 for IL-6 and *r* = 0.88, *P* < .001 for TNF-α). Furthermore, IP-10 and eotaxin were detectable but diminished in CRPS1, whereas detectable amounts of IL-10 were similar in involved and noninvolved extremities. Multiplex bead array assays are useful systems to establish the involvement of cytokines in inflammatory processes by measurements in blister fluids of CRPS1. Ten representative cytokines were detectable. However, detection levels and amounts measured are at least 3 times higher in the multiplex-25 array assay than in the ELISA assays used simultaneously for the measurement of cytokines.

## INTRODUCTION

Complex regional pain syndrome type 1 (CRPS1), also known as reflex sympathetic dystrophy (RSD), is a debilitating painful disease in an extremity that is characterized by signs of allodynia and hyperalgesia, as well as vasomotor, sudomotor, and motor trophic signs and symptoms. In general the disease persists in one extremity [1, 2]. The diagnosis of CRPS1 is mainly based on clinical observation [3, 4], for which international research criteria have been determined [5]. Although some patients develop CRPS1 after an inciting event (trauma or surgery in the hand, foot, or knee), the origin of this invalidating disease remains unknown. Subgroups of CRPS1 patients are described in whom either vasomotor signs, neuropathic pain, or all signs of inflammation are prominent factors [6]. Studies on the underlying mechanisms of this disease have ranged from the effects of physiotherapy to pharmaceutical intervention and from biological active mediators to genetic mapping. During the initial stage of the disease most symptoms, such as oedema, redness, loss of function, and temperature changes [7], suggest a local inflammatory process [8]. Therefore we subsequently investigated the involvement of inflammatory mediators during the initial stage of this disease and showed that the cytokines interleukin-6 (IL-6) and tumour necrosis factor α (TNF-α) were significantly increased in the affected hand or foot [9], which was confirmed by other markers of inflammation [10]. Most treatments of CRPS1 are not evidence based. The patient-dependent choice of either physical therapy, pharmaceutical intervention, or unconventional alternative medicine is still a matter of debate [8, 11]. Targeted treatment with anti-TNF (Infliximab) seems, however, to be successful in patients with confirmed signs of inflammation [12].

In all our recent studies, skin blister fluids showed elevated amounts of IL-6 and TNF-α as a measure of local inflammation intensity. Due to the limited amount of fluid, however, in the same sample we were only able to measure 2 or 3 different mediators separately. Therefore the present study aimed to confirm the involvement of inflammatory processes underlying CRPS1 by measuring a large variety of cytokines simultaneously in the same small blister fluid sample.

Until now commercially available enzyme-linked immunosorbent assay (ELISA) kits are used to measure levels of cytokines in biological samples. Most of these kits require a two-fold diluted sample volume of 100 μL. Therefore, to examine a number of different classes of cytokines, volumes of more than a few hundred μL should be available, otherwise dilutions need to be made. However, this process of dilution could result in values that are below the detectable standard. The simultaneous measurement of a number of cytokines in a single sample using a new developed microbead-based flow cytometry system (Luminex) enables to detect of cytokines in small volume samples of human biological material [13].

Successful measurement of six Th1/Th2 cell distinguishing cytokines (interferon-γ (IFN-γ), TNF-α, IL-2, IL-4, IL-5, and IL-10) has been reported in a single sample of human tears obtained from allergic patients [14, 15], and in plasma from children with neonatal sepsis; in these newborn infants, in the same samples the contribution of inflammatory cytokines (IL-1β, IL-6, IL-8, IL-10, IL-12, and TNF-α) was also evaluated [16]. This so-called inflammation panel was also used for the simultaneous measurement of cytokines in tracheal aspirates after mechanical ventilation [17].

Here we report on the simultaneous detection of 25 cytokines in blister fluids obtained from both the involved and the noninvolved (contralateral) extremities of CRPS1 patients. This is the first study in which such a large number of inflammatory cytokines, Th1/Th2 distinguishing cytokines, and chemokines have been investigated in human skin blister fluids.

## METHODS AND MATERIALS

### Patients and blister fluids

For this study 22 patients (4 males, 18 females; mean age 52 ± 8.2 (SD) years) were selected, with a mean duration of the disease of 2.75 ± 1.25 (SD) years, all being in the intermediate phase. During CRPS1 we in general distinguish four different disease phases. Stage I is defined as the warm or hypertrophic phase, stage II is defined as the intermediate phase, stage III is defined as the cold or early chronic phase, and at last in stage IV the definite chronic phase corresponds to atrophic signs, dystonia, and per definition stabilization of the disease or, in rare instances, to healing [8–12, 18].

All 22 patients were characterized using the impairment sum score (ISS, according to Oerlemans et al [7]). At the time of the study this was 38 ± 16.6 (SD) on a scale of 0–100, indicating a medium disease activity. This score was calculated based on differences in skin surface temperature, volume of oedema, quantity of pain (visual analogue scale), intensity of pain (McGill Pain Questionnaire), and motor function (as active range of motion).

Blisters were induced by means of a suction method [9, 10]. A 3-hole (5 mm diameter per hole) skin suction chamber was positioned on the skin of the upper extremity, on the dorsal side of the involved hand and the flexor side of the noninvolved forearm.

A vacuum of 300 mm Hg negative pressure was applied with an Atmoforte 350A aspirator pump (ATMOS MedizinTechnik, Lenzkirch, Germany), which was reduced after 15 minutes to 250 mm Hg and again, 15 minutes later, reduced to 200 mm Hg. This negative pressure was maintained until blisters containing sufficient fluid had been developed, but not longer than 2.5 hours. The contents of the blisters were punctured and pooled from each side into a 1.5 mL Eppendorf conical polypropylene tube and centrifuged for 5 minutes at 1600 x*g*. The mean recovery of supernatants from control blisters was 173 ± 21 (± SEM) μL fluid, and 168 ± 17μL blister fluid from the CRPS1 side. All samples were stored in 1 mL conical polypropylene tubes at −80°C until analysis [9].

In these blister fluid samples IL-6 and TNF-α were analyzed separately by ELISA and a set of 25 cytokines were analyzed simultaneously using the Luminex system and the multiplex-25 array assay from Biosource.

### Enzyme-linked immunosorbent assays

Blister samples were diluted 4-fold in appropriate calibrator diluent assay buffer for the direct measurement of cytokines. Cytokine assays were performed following the manufacturers protocol (Pelikine human ELISA compact kits for IL-6 (M1906) and TNF-α (M1920), Sanquin, Amsterdam, The Netherlands). The standard curve ranges and mean calculated zero signal plus 3 SD for IL-6 were 0–450 pg/mL and 0.2 pg/mL, respectively; and for TNF-α 0–1000 pg/mL and 1 pg/mL, respectively. The requested solutions were provided with the ELISA compact kits and additional toolkits (Pelikine-Tool set (M1980), Sanquin, Amsterdam, The Netherlands).

In brief, the ELISA procedure (performed at room temperature) was as follows: The wells of a 96-well plate were precoated overnight with 100 μL of coating antibody, diluted 1 : 100 with coating buffer (0.1 M carbonate/bicarbonate). Thereafter the wells were washed 5 times with 400 μL of phosphate buffered saline (PBS) containing 0.005% Tween and then blocked with 200 μL of blocking buffer (1 : 20 diluted in PBS) for 1 hour on a shaker. After washing the plate five times with washing buffer, 100 μL of unknown blister fluid samples (diluted 1 : 4 in assay dilution buffer) or standards were pipetted into the wells. The plate was incubated for 1 hour on a shaker. After washing the plate five times with washing buffer, 100 μL of biotinylated antibody (diluted 1 : 100 in assay dilution buffer) was pipetted into the wells and incubated 1 hour on the shaker. After washing the plate, the wells were incubated 30 minutes on a shaker with 100 μL of streptavidin-HRP conjugate (diluted 1 : 10,000 in assay dilution buffer). Thereafter the plate was washed for the last time with washing buffer and incubated with 100 μL of tetramethylbenzidine substrate solution. The reaction was stopped after 30 minutes with 100 μL of stop solution (1.8 M_2_SO_4_). The absorbance per well was measured at 450 nm with a Medgenix ELISA reader. Sample concentrations were calculated using the appropriate standard calibration lines and the Softmax software of the reader.

### Multiplex-25 bead array assay

The human cytokine multiplex-25 bead array assay kit for Luminex was purchased from Biosource (Nivelles, Belgium). This kit comprises all components necessary for the whole assay procedure to be fulfilled within approximately 5 hours hands-on time.

The following cytokines could be measured:

- *inflammatory panel*: GM-CSF (granulocyte-macro-phage colony-stimulating factor), IL-1β, IL-1RA (interleukin-1 receptor antagonist), IL-6, IL-8, TNF-α;
- *Th1/Th2 panel*: IFN-γ, IL-2, IL-2R, IL-4, IL-5, IL-10;
- *cytokine II panel*: IFN-α, IL-7, IL-12p40/p70, IL-13, IL-15, IL-17;
- *chemokine panel*: eotaxin, IP-10 (interferon-γ inducing protein, 10 kDa), MCP-1 (monocyte chemotactic protein), MIP-1α (macrophage inflammatory protein), MIP-1β, MIG (monokine induced by γ-interferon), RANTES (regulated upon activation normal T cell expressed and secreted).

Standard curves for each cytokine (in duplicate) were generated by using the reference cytokine concentrations supplied in this kit. Blister samples were diluted 4-fold in appropriate assay diluent. The assay was performed in a 96-well filter plate, using all the assay components provided in the kit. All incubation steps were performed at room temperature and in the dark to protect the beads from light.

In brief, the following procedure was performed: firstly, the filter plate was prewetted with 200 μL of working washing solution and then this solution was aspirated from the wells using a vacuum manifold. The beads (25 μL) were pipetted into each well and thereafter the filter plate wells were washed two times with washing buffer using the vacuum manifold. Incubation buffer (50 μL) and 1 : 4 diluted blister fluid samples or standards (50 μL) were pipetted into the wells and incubated for 2 hours with the beads. Thereafter the wells were washed using the vacuum manifold and detector antibody conjugated to biotin (diluted 1 : 10 with biotin diluent) was added. After incubation for 1 hour, beads were washed again followed by an incubation of 30 minutes with streptavidin conjugated to the fluorescent protein, R-phycoerythrin (Streptavidin-RPE, diluted 1 : 10). After washing to remove the unbound Streptavidin-RPE, the beads (minimum of 50 beads per cytokine) were analyzed in the Luminex 100 instrument (Applied Cytometry Systems, Dinnington, UK), which monitored the spectral properties of the beads while simultaneously measuring the amount of fluorescence associated with R-phycoerythrin. Raw data (mean fluorescence intensity, MFI) were analyzed using StarStation software (Applied Cytometric Systems, Dinnington, UK).

### Statistical analysis

**Figure 1:**
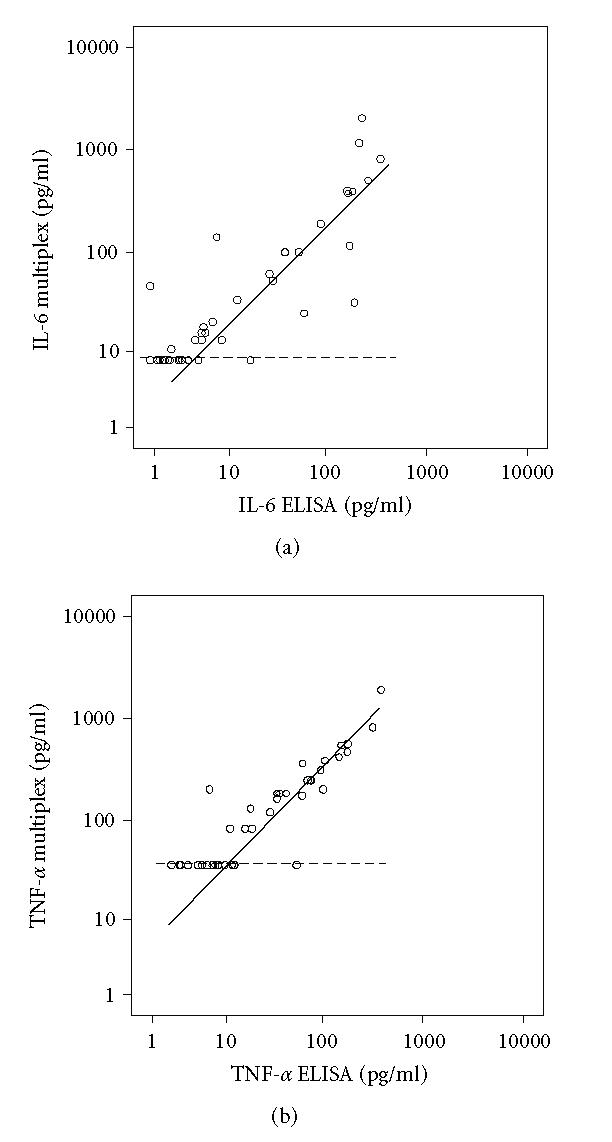
Regression curves of 44 samples of blister fluid obtained from 22 CRPS1 patients, both from the involved and the noninvolved extremity. Values calculated in pg/mL were plotted on logarithmic scales. Regression lines were calculated taking into account the left-censored values due to detection limits as described in the statistical methods. Dotted lines indicate detection levels of the multiplex-25 cytokine assay. (a) Regression curve of IL-6 data from the multiplex-25 cytokine assay and the ELISA kit (*r* = 0.85, *P* < .001), (b) regression curve of TNF-α data from the multiplex-25 cytokine assay and the ELISA kit (*r* = 0.88, *P* < .001).

All cytokines showed a skewed distribution. Comparison of paired samples (CRPS1 versus noninvolved extremity) was performed with the paired *t* test after logarithmic transformation of the data obtained from measurements in blister fluids. In case of values below the detection limit, the outcome was set at the detection limit and the paired sample *t* test with adjustment for these left-censored values was performed using STATA software (CNREG procedure). The same method was used to assess the assumed linear relation shown in Figure 1. Correlation coefficients were determined by the Spearman's test for untransformed data.

## RESULTS

To our knowledge we are the only clinical investigators reporting on cytokine levels in blister fluids obtained from CRPS1 patients [9, 10, 19]. Therefore we searched for data obtained from artificial blisters in immunological skin diseases in order to compare detection ranges (Table 1).

*ELISA*

Both IL-6 and TNF-α were measured in blister fluid samples of 22 CRPS1 patients, obtained from both the involved and the noninvolved extremity. Standards were measured in duplicate for 8 data points including a zero standard. Standard curves were plotted through a four-parameter logistic curve fitting. *R*-squared values were 0.999 and 1.00, respectively. Calculated levels are presented in Table 2. Because cytokines displayed a not normally distributed set of data, the median and the ranges are presented. Interleukin-6 and TNF-α were significantly increased at the CRPS1 side (paired sample test).

*Multiplex*

Twenty-five cytokines were measured in the same blister fluid samples of the 22 CRPS1 patients as indicated in the previous section “ELISA.” Cytokine-specific single beads (25 different bead populations) were identified through sequential gating on doublet discriminator signal and intrinsic bead dye (red versus infrared) excluding bead aggregates and debris. The amount of cytokine was measured as mean fluorescence intensity (MFI) of the Streptavidin-RPE signal on the outside of the beads from a minimum of 50 beads per cytokine. Standards were measured in duplicate for 9 data points including a zero standard.

Standard curves were plotted through a four- or five-parameter logistic curve fitting. All *R*-squared values were between 0.99 and 1.00, except for IL-7 (0.968).

In blister fluid from the “*inflammatory panel*” IL-1RA, IL-6, IL-8, and TNF-α were detectable, from the “*cytokine II panel*” IL-12p40 was detectable and from the “*chemokine panel*” MCP-1 and MIP-1β were detectable and all were increased in CRPS1 affected extremities.

Furthermore, from the “*chemokine panel*” IP-10 and eotaxin were detectable and diminished in CRPS1, whereas from the “*Th1/Th2 panel*” detectable amounts of IL-10 were similar in both extremities (Table 3).

*Statistical considerations*

An analysis by using nonparametric statistics (Wilcoxon's signed rank test), with outcomes set at the lower limit of detections in case of values below this limit, resulted in similar *P*-values for all parameters. We did not adjust for multiple comparison tests because our study had an exploratory character.

*Comparison of the two methods*

Levels of IL-6 and TNF-α measured by ELISA and by the multiplex-25 bead array assay were highly correlated (*r* = 0.85, *P* < .001 for IL-6, Figure 1(a), and *r* = 0.88, *P* < .001 for TNF-α, Figure 1(b)).

In the multiplex-25 bead array assay for IL-6 17 of 44 samples were not detectable (≤ 8 pg/mL), whereas for TNF-α 20 of 44 samples were not detectable (≤ 36 pg/mL). In the IL-6 ELISA only one sample was below the detection level.

*Correlations of multiplex-25 measured cytokines*

**Figure 2:**
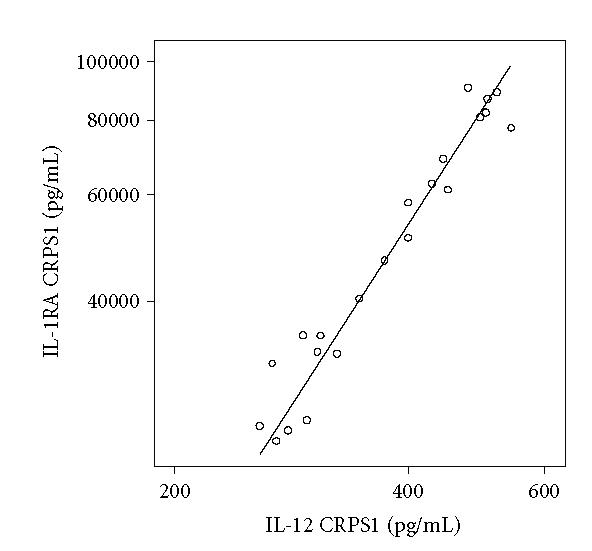
Example of a regression curve between concentrations of IL-1RA and IL-12 in 22 blister fluid samples taken from the CRPS1 extremity (correlation coefficient 0.97, *P* < .001), measured by the multiplex-25 bead array assay. Values are calculated in pg/mL and plotted on a logarithmic scale.

The cytokines IL-6, IL-8, IL10, IL-12, TNF-α, MIP-1β, and MCP-1 were significantly correlated with each other (Table 4), whereas IL-12p40 was only (highly) correlated with IL-1RA (Figure 2).

## DISCUSSION

This study investigated the involvement of inflammatory mediators in CRPS1 represented by a large variety of cytokines simultaneously measured in one small blister fluid sample. Use of the new multiplex-25 bead array assay allowed to determine 10 cytokines in blister fluid samples, considerably above the relatively high detection levels of these integrated cytokine assays.

*Inflammatory panel*

In our earlier observations of mediators in blister fluid of CRPS1 patients (reflecting inflammation at the affected extremity), we decided to measure both IL-6 and TNF-α as representative markers of inflammation [9, 10, 19]. At that time the amounts of cytokines we found were within the ranges reported for other (mainly dermatological) diseases (Table 1). The present results obtained after simultaneous measurement of cytokines by the multiplex-25 array assay were (although 2-3 times higher) in the same range. In those 44 blister fluid samples high correlations were found between data obtained from ELISA measurements and the multiplex-25 bead array assays (Figures 1(a) and 1(b)).

In addition to these findings, significant amounts of other (pro-) inflammatory cytokines were detectable in blister fluid by the multiplex-25 bead array assay, namely, IL-8 and IL-12p40/p70. Furthermore, relatively high amounts of IL-1RA were found. Amounts of IL-1RA were comparable to those found by Blaha et al [20].

Using the multiplex-25 bead array assay, GM-CSF was not detectable. This was, however, also the case in blister fluid of patients with bullous pemphigus (BP), a chronic autoimmune blistering disease, in which GM-CSF was not detectable (< 5 pg/mL) by ELISA [21].

*Th1/Th2 panel*

In the present study we concluded that the cytokines IFN-γ, IL-2, IL-2R, IL-4, IL-5, and IL-10 normally involved in the Th1/Th2 pathways were negligible, because all calculated data were around or beneath the detection limits of the multiplex-25 bead array assay.

Detectable amounts of IL-4 and IL-10 (Table 1) and of soluble IL-2R have been measured in the blister fluid of patients with toxic epidermal necrolysis, a disease in which the early participation of activated CD8^+^ T lymphocytes play an important role [22].

*Chemokines*

Eotaxin and IL-5 are representative chemotactic cytokines to study the activation of skin-homed eosinophils, which in general represent allergic reactions [23]. In pemphigoid gestationis, a rare autoimmune bullous disease of late pregnancy, both markers are significantly increased in blister fluid [24]. In another study, elevated levels of both eotaxin and IL-5 in blister fluid of BP were found, suggesting tissue eosinophilia [25].

In the present study IL-5 was not detectable, although low detection ranges were achieved. On the contrary, eotaxin was detectable at levels > 12 pg/mL blister fluid, but was surprisingly decreased in CRPS1 blister fluid. Therefore, we concluded that allergic reactions do not play an important role in CRPS1.

In our study, both MCP-1 and MIP-1β were present in blister fluid in significant amounts and were increased in CRPS1 blisters in comparison with noninvolved blister samples, suggesting an ongoing involvement of activated monocytes and macrophages. In blisters generated in skin of chronic ambulatory peritoneal dialysis patients, however, MCP-1 concentrations in this interstitial fluid were not related to the intensity of the inflammation [26, 27].

*Source of cytokines*

The involvement, cellular sources and most prominent effects of cytokines in BP, partly detected in blister fluids, have been reviewed extensively [28, 29]. In our observations, a number of detectable mediators measured at the CRPS1 side were correlated individually, except for IL-1RA, eotaxin, and IP-10 (Table 4). Our data suggest that detectable mediators have been generated by a homogenous cell population. Because T-cells apparently are not involved, the most likely candidates are monocytes, macrophages, and possibly fibroblasts. The main products generated by these cells are IL-6, IL-8, IL-10, IL-12, and TNF-α. Apparently, skin mast cells are also involved, as reflected by increased amounts of tryptase in CRPS1 blister fluid [10]. The main cytokine produced by mast cells is TNF-α.

The amount of cytokines IL-8, IL-6, MCP-1, GM-CSF, TNF-α, and MIP-1β secreted by human epithelial cells from the female reproductive tract was recently assessed by Luminex bead array analysis [30]. The main products found were IL-8 and IL-6, but these were 100-fold higher than those of GM-CSF, TNF-α, and MIP-1β. Therefore, regarding the distribution of our data, it is unlikely that epithelial cells contributed to the levels of cytokines found in blister fluid of CRPS1 patients.

*Sensitivity of multiplex-25 bead array assay*

The sensitivity of multiplex bead array assays for the detection of soluble cytokines and the quantitative values from several manufacturers have been compared for serum samples [31]. Bead array and ELISA values appeared to be comparable between the manufacturers. The minimal detection range for the Biosource kit was comparable with the R&D Systems assay kit, but about 2-fold and 5-fold higher than kits from Bio-Rad and LINCO Research, respectively. The simultaneous measurement of 15 human cytokines (Bio-Plex system from Bio-Rad) in a single sample of cultured peripheral blood mononuclear cells compared with regular ELISA kits (purchased from a number of manufacturers), resulted in high correlation coefficients ranging from 0.75 to 0.99 [32]. Our comparison between the multiplex-25 bead array assay and ELISAs for IL-6 and TNF-α also revealed high correlation coefficients, ranging from 0.85 to 0.88 (Figure 1).

The high detection levels in the present study were partly caused by the 4-fold dilutions needed to enable separate determinations by ELISA techniques. Generally, at least 50 μL will be recovered per blister evoked by suction. After a 2-fold dilution in assay matrix buffer a duplicate measurement by the multiplex bead array assay could be performed. Then detection levels will be more acceptable; however, they will still be 3-fold (or even more) increased compared with the commonly used ELISAs. In case of paired sample measurements (involved versus noninvolved) or treatment-affected paired sample measurements, these shortcomings are acceptable for the selected cytokine panels as demonstrated by the results of this study.

We failed to detect substantial amounts of protein in blister fluid of at least 15 selected cytokines assayed in this multiplex-25 array system, although the detection of some of these cytokines (such as IL-1β, IL-2, IL-5, IL-7, IL-15, IFN-γ, and RANTES) has been realized in blister fluid using commonly available ELISA kits which are more sensitive [28]. Based on these ELISA derived data, the levels of cytokines detectable in blister fluids taken from a variety of diseases generally were above our detection limits. Therefore, we concluded that these cytokines are not prominent mediators involved in CRPS1.

## CONCLUSION

Based on our findings, routine application of a multiplex-25 bead array assay to detect representative cytokines in blister fluids would not be advisable. The use of this system is advisable for investigational purposes or for diagnosis based on selected cytokines from relevant literature. We therefore propose that a selection of two or three representatives from each panel (the inflammatory cytokines panel, the Th1/Th2 cytokines panel, and the chemokines panel) would be sufficient to indicate the activity of the CRPS1 disease. During the course of the disease this selected panel could also be used to indicate the effectiveness of therapeutic intervention. Based on our data and the selection made by Fahey et al [30], we suggest to include at least IL-6, IL-8, TNF-α, MCP-1, MIP-1β, IL-10, and IL-12 in that investigation panel.

Future research using blister fluid should also focus on standardization of the blister techniques and the warranted inclusion of control samples, either from noninvolved tissue or from healthy volunteers.

## Figures and Tables

**Table 1:** Literature overview of cytokine levels in blister fluid measured by ELISA. Data are medians or otherwise means (indicated by ^m^). ND: not determined.

Disease	Refs	Extremity	IL-1β	IL-6	IL-8	TNF-α	IL-4	IL-10
			(pg/mL)	(pg/mL)	(pg/mL)	(pg/mL)	(pg/mL)	(pg/mL)

Complex regional	9,10	involved	≤ 2	54	—	31	—	—
pain syndrome		noninvolved	≤ 2	6	—	8	—	—
Psoriasis	33,34	involved	122	1683	—	145	—	—
		noninvolved	≤ 3	121	—	9	—	—
Psoriasis	35	involved	—	870	—	195	—	—
		noninvolved	—	423	—	84	—	—
Epidermal	36	involved	—	66	10	—	—	33
necrolysis		noninvolved	—	ND	ND	—	—	ND
Bullous	21,37	involved	73^m^	245^m^	—	—	9	54
pemphigus		noninvolved	2^m^	16^m^	—	—	≤ 4	≤ 5
Bullous	38	involved	—	—	—	—	—	73
pemphigus		noninvolved	—	—	—	—	—	ND
Pemphigus	39	involved	—	—	—	—	—	186^m^
vulgaris		noninvolved	—	—	—	—	—	ND

**Table 2:** Cytokine levels in blister fluids from 22 patients with complex regional pain syndrome measured by ELISA. Blister fluids were diluted 4-fold in matrix buffer. Lowest detectable level: lowest detectable standard which significantly differs from zero standard (experimentally determined).

	lowest detectable	levels in blister fluid median (range) in pg/mL		
ELISA	level (pg/mL)	noninvolved	CRPS1	*P*-value

*Inflammatory panel*				

IL-6	0.2	2.7 (≤ 0.8–191)	38 (≤ 0.8–346)	0.002
TNF-α	0.5	10.3 (2.1–315)	48 (2.8–381)	0.006

**Table 3:** Cytokine levels in blister fluids from 22 patients with complex regional pain syndrome measured by multiplex-25 bead array assay. Blister fluids were diluted 4-fold in matrix buffer. Lowest detectable level: lowest detectable standard which significantly differs from zero standard (experimentally determined). *P*-values: nt: not tested, because all measured outcomes were below detection level.

	lowest detectable	levels in blister fluid median (range) in pg/mL		
25-plex	level (pg/mL)	noninvolved	CRPS1	*P*-value

*Inflammatory panel*				

GM-CSF	11	all ≤ 44	all ≤ 44	nt
IL-1β	12	all ≤ 48	all ≤ 48	nt
IL-1RA	50	35940	48894	< .001
		(12665–67549)	(23393–90714)	
IL-6	2	≤ 8 (≤ 8–100)	100 (≤ 8–2055)	.001
IL-8	7	≤ 28 (≤ 28–301)	46 (≤ 28–519)	.006
TNF-α	9	≤ 36 (≤ 36–829)	195 (≤ 36–1923)	.013

*Th1/Th2 panel*				

IFN-γ	3	all ≤ 12	all ≤ 12	nt
IL-2	4	all < 16	all ≤ 16	nt
IL-2R	30	all ≤ 120	all ≤ 120	nt
IL-4	2	all ≤ 8	all ≤ 8	nt
IL-5	2	all ≤ 8	all ≤ 8	nt
IL-10	4	20 (≤16 –51)	21 (≤ 16–50)	.336

*Cytokine II panel*				

IFN-α	10	all ≤ 40	all ≤ 40	nt
IL-7	28	all ≤ 112	all ≤ 112	nt
IL-12p40	4	325 (192–540)	386 (256–542)	.007
IL-13	3	all ≤ 12	all ≤ 12	nt
IL-15	6	all ≤ 24	all ≤ 24	nt
IL-17	6	all ≤ 24	all ≤ 24	nt

*Chemokine panel*				

Eotaxin	3	29 (15–54)	24 (≤ 12–55)	.009
IP-10	3	48 (24–185)	37 (≤ 12–137)	.025
MCP-1	3	297 (126–1570)	579 (188–4415)	.002
MIP-1α	10	all ≤ 40	all ≤ 40	nt
MIP-1β	10	199 (116–450)	290 (135–557)	.001
MIG	12	all ≤ 48	all ≤ 48	nt
RANTES	10	all ≤ 40	all ≤ 40	nt

**Table 4:** Nonparametric correlations of cytokines in blister fluid from CRPS1 hand. *P*-values: ^a^ < .001, ^b^ < .005, ^c^ < .01, ^d^ < .02, ^e^ < .05.

	IL-8	IL-10	IL-12	TNF-α	MIP-1β	MCP-1

IL-1RA	—	—	0.94^a^	—	—	—
IL-6	0.85^a^	0.56^c^	0.51^d^	0.78^a^	0.72^a^	0.88^a^
IL-8	—	0.63^b^	0.44^e^	0.93^a^	0.76^a^	0.88^a^
IL-10	—	—	—	0.56^c^	0.62^b^	0.59^b^
IL-12	—	—	—	0.48^e^	0.50^d^	0.47^e^
TNF-α	—	—	—	—	0.67^b^	0.81^a^
MIP-1	—	—	—	—	—	0.68^e^
